# Metabolic alterations by indoxyl sulfate in skeletal muscle induce uremic sarcopenia in chronic kidney disease

**DOI:** 10.1038/srep36618

**Published:** 2016-11-10

**Authors:** Emiko Sato, Takefumi Mori, Eikan Mishima, Arisa Suzuki, Sanae Sugawara, Naho Kurasawa, Daisuke Saigusa, Daisuke Miura, Tomomi Morikawa-Ichinose, Ritsumi Saito, Ikuko Oba-Yabana, Yuji Oe, Kiyomi Kisu, Eri Naganuma, Kenji Koizumi, Takayuki Mokudai, Yoshimi Niwano, Tai Kudo, Chitose Suzuki, Nobuyuki Takahashi, Hiroshi Sato, Takaaki Abe, Toshimitsu Niwa, Sadayoshi Ito

**Affiliations:** 1 Division of Nephrology, Endocrinology and Vascular Medicine, Tohoku University Graduate School of Medicine, Sendai, 980-8574, Japan; 2 Division of Clinical Pharmacology and Therapeutics, Tohoku University Graduate School of Pharmaceutical Sciences, Sendai, 980-8578, Japan; 3 Division of Integrative Renal Replacement Therapy, Tohoku University Graduate School of Medicine, Sendai, 980-8574, Japan; 4 Division of Nephrology and Endocrinology, Tohoku Medical and Pharmaceutical University, Sendai, 981-8512, Japan; 5 Department of Integrative Genomics, Tohoku Medical Megabank Organization, Tohoku University, Sendai, 980-8573, Japan; 6 Innovation Center for Medical Redox Navigation, Kyushu University, Fukuoka, 812-8582, Japan; 7 Laboratory for Redox Regulation, Tohoku University Graduate School of Dentistry, Sendai, 980-8575, Japan; 8 Primetech Co. Ltd., Tokyo, 112-0002, Japan; 9 Shubun University, Ichinomiya, 491-0938, Japan

## Abstract

Sarcopenia is associated with increased morbidity and mortality in chronic kidney disease (CKD). Pathogenic mechanism of skeletal muscle loss in CKD, which is defined as uremic sarcopenia, remains unclear. We found that causative pathological mechanism of uremic sarcopenia is metabolic alterations by uremic toxin indoxyl sulfate. Imaging mass spectrometry revealed indoxyl sulfate accumulated in muscle tissue of a mouse model of CKD. Comprehensive metabolomics revealed that indoxyl sulfate induces metabolic alterations such as upregulation of glycolysis, including pentose phosphate pathway acceleration as antioxidative stress response, via nuclear factor (erythroid-2-related factor)-2. The altered metabolic flow to excess antioxidative response resulted in downregulation of TCA cycle and its effected mitochondrial dysfunction and ATP shortage in muscle cells. In clinical research, a significant inverse association between plasma indoxyl sulfate and skeletal muscle mass in CKD patients was observed. Our results indicate that indoxyl sulfate is a pathogenic factor for sarcopenia in CKD.

Sarcopenia is a muscle wasting syndrome characterized by progressive loss of generalized skeletal muscle mass and strength with a risk of physical disability and poor quality of life^1^,^2^. In chronic kidney disease (CKD), sarcopenia is prevalent and particularly prominent among uremic patients with end-stage renal disease requiring dialysis^3^,^4^. Thus, CKD-associated sarcopenia is also called “uremic sarcopenia”^5^. Uremic sarcopenia is a serious clinical problem because of its strong association with increased morbidity and mortality^5^.

Renal dysfunction results in the accumulation of various uremic toxins in the circulation^6^. Because accumulated uremic toxins show deleterious and cytotoxic effects^7^, they contribute to various complications, such as hypertension, cardiovascular disease, neuro disorders, and bone disorders that appear with CKD^8^,^9^,^10^,^11^. The accumulation of uremic toxins has been proposed as a contributing factor for uremic sarcopenia; however, the physiological relationship remains unclear.

Accumulation of uremic toxins causes metabolic alterations, such as metabolic acidosis, that are prevalent in advanced CKD patients^12^. Metabolic acidosis promotes muscle protein wasting by increasing protein degradation and reducing protein synthesis^13^,^14^,^15^. Protein degradation signaling in muscle cells leads to impairment of carbohydrate oxidation and consequently contributes to sarcopenia^16^. Therefore, metabolic alterations in muscle cells have an important role in uremic sarcopenia.

Indoxyl sulfate (IS) is an extensively researched compound among uremic toxins^17^,^18^,^19^,^20^. IS is an organic anion produced from indole derived from dietary tryptophan^21^. IS has been reported to show strong nephro-vascular toxicity via disruption of redox homeostasis by oxidative stress and downregulation of mitochondrial metabolism. It is therefore considered a critical factor for the progression of CKD^11^,^22^,^23^. However, the adverse effects of IS on skeletal muscle are not well known.

In this study, we hypothesized that the accumulation of uremic toxins is a trigger for skeletal muscle reduction and contributes to uremic sarcopenia in CKD. To verify this hypothesis and elucidate the mechanism, we examined the effects of the representative uremic toxin IS on skeletal muscle, with a focus on alterations in cellular metabolism and mitochondrial functions. Because IS shows multi-faced effects on the cells such as redox homeostasis disruption, metabolic changes, and mitochondrial dysfunction, we comprehensively analyzed the effect of IS including metabolomic alteration examined by capillary electrophoresis mass spectrometry (CE-MS), mitochondrial function examined by extracellular flux measurements, and the expression of mRNA and protein related to causative oxidative stress response pathways. Additionally, we investigated the association between plasma IS and muscle condition in CKD patients. Our findings suggest that IS contributes to uremic sarcopenia through induction of metabolic alterations such as excess antioxidative response and mitochondrial disorders and inhibition of anabolism signaling in skeletal muscle.

## Results

### Histological changes and accumulation of IS in skeletal muscle of CKD mice

**Figure 1:**
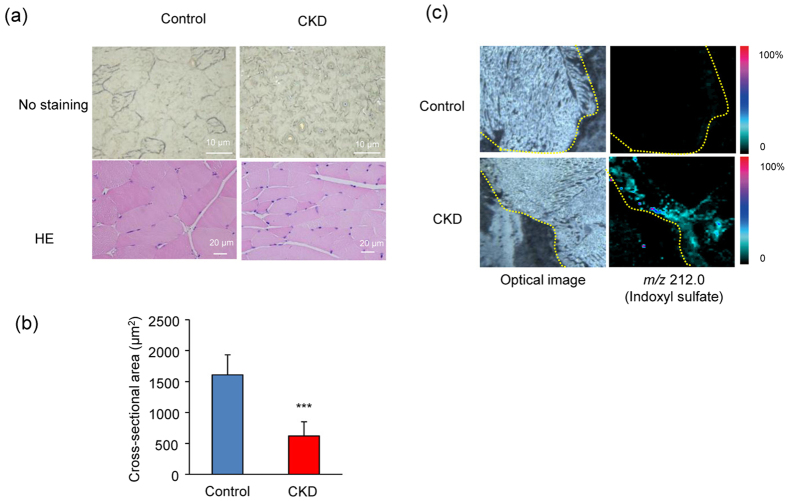
Morphological alterations of muscle tissues and IS uptake in CKD model mice. **(a)** Histological changes in femoral muscles of control and adenine-induced CKD model mice (CKD). Upper panels are optical images without staining of femoral muscle frozen sections. Lower panels are hematoxylin and eosin (HE) staining of anterior tibia muscle paraffin sections. **(b)** Comparison of cross-sectional area of anterior tibia muscle between control and CKD model mouse. Values are mean ± SD (*n* = 30), ***p < 0.0001, difference with control by Student *t*-test. Data are mean ± SD. **(c)** Mass spectrometry imaging-based cellular uptake of IS into adenine-induced CKD model mouse. Tissue sections from femoral muscle from control and adenine-induced CKD model mouse shown. Molecular image of *m/z* 212.0 represent amount and distribution of IS.

To evaluate changes in skeletal muscle in uremic sarcopenia, we examined skeletal muscle of a mouse model of adenine-induced CKD that shows significant increases in circulating IS^24^. Histological changes in skeletal muscle are shown in Fig. 1. The morphological structure of skeletal muscle in CKD mice showed marked alignment dissolution, and was significantly (p < 0.001) smaller compared with that of control mice (Fig. 1a,b), suggesting that CKD mice had atrophic skeletal muscle.

To investigate the effects of accumulated circulating IS on skeletal muscle, we analyzed intramuscular IS concentrations using imaging mass spectrometry. Cellular IS was determined at an ion signal of *m/z* 212 (Fig. 1c). The signal corresponding to IS was strongly detected in skeletal muscle of CKD mice, and was slightly detected in skeletal muscle of control mice. These findings indicated that accumulated IS in the circulation was imported into skeletal muscle cells in CKD mice and represented atrophic skeletal muscle.

### Accumulation of IS and its effects on proliferation in C2C12 cells

**Figure 2:**
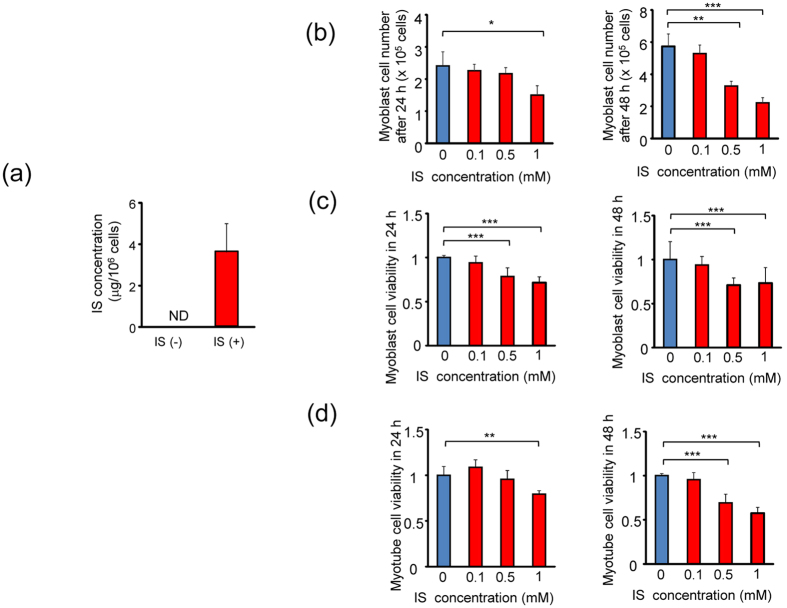
IS inhibits cell proliferation in myoblast and myotube cells. **(a)** Uptake of IS in C2C12 myotube cells. C2C12 myotube cells were incubated with and without 1 mM IS for 24 h at 37 °C. Amount of IS in cellular extracts shown. Values are mean ± SD (*n* = 4). **(b)** C2C12 myoblast cells were exposed to IS at indicated concentrations for 24 and 48 h, followed by cell counting (*n* = 3). **(c)** C2C12 myoblast cells exposed to IS at indicated concentrations for 24 and 48 h, followed by MTT assay (*n* = 8). **(d)** C2C12 myotube cells exposed to IS at indicated concentrations for 24 and 48 h, followed by MTT assay (*n* = 8). Data are mean ± SD, *p < 0.05, **p < 0.01, ***p < 0.0001, difference with control (IS: 0 mM) by Tukey-Kramer’s test.

To investigate the mechanism behind IS in skeletal muscle of uremic sarcopenia, we used the mouse myoblast cell line C2C12 and differentiated myotubes. We examined the cellular import of IS into C2C12 myotubes using liquid chromatography–tandem mass spectrometry (LC-MS/MS) (Fig. 2a). Although cellular IS was not detected in C2C12 myotubes under normal culture conditions, culture with IS-containing medium increased intracellular IS up to approximately 4 μg/1 × 10^6^ cells, suggesting that extracellular IS was imported into cultured myotubes.

We then examined the toxic effects of IS on skeletal muscle using C2C12 cells. Under control culture conditions, C2C12 myoblast cells grew up to 2.4- and 5.7-fold in 24 h and 48 h, respectively. However, exposure to 1 mM IS significantly inhibited cell proliferation (Fig. 2b). Cell viability for both C2C12 myoblast cells and myotubes was significantly lower under IS-treated conditions compared with control conditions in a concentration-dependent manner (Fig. 2c,d). These results indicated that IS exerted a toxic effect on skeletal muscle cells by inhibiting cell proliferation and viability.

### IS induces metabolic alterations in myotubes

**Figure 3:**
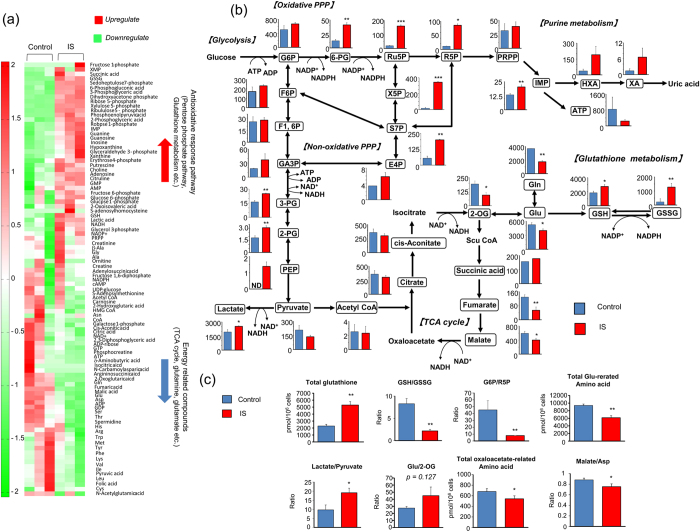
Metabolic response of C2C12 myotube cells upon exposure to IS. (**a**) Metabolic pathway enrichment analysis. (**b**) Shown are relative changes of metabolite levels in glycolysis, PPP, purine metabolism, and TCA cycle comparing non-treated (*blue*) and IS-treated (*red*) C2C12 cell extracts. Data are mean ± SD (*n* = 3), *p < 0.05, **p < 0.01, ***p < 0.0001, difference with control by unpaired t-test. Vertical axis shows absolute value (pmol/10^6^ cells). G6P: glucose 6-phosphate; F6P: fructose 6-phosphate; F1,6P: fructose 1,6-diphosphate; GA3P: glyceraldehyde 3-phosphate; 3-PG: 3-phosphoglyceric acid; 2-PG: 2-phosphoglyceric acid; PEP: phosphoenolpyruvate; 6-PG: 6-phosphogluconate; Ru5P: ribulose 5-phosphate; PRPP: phosphoribosyl pyrophosphate; X5P: xylulose 5-phosphate; S7P: sedoheptulose 7-phosphate; E4P: erythrose 4-phosphate; IMP: inosine monophosphate; HXA: hypoxanthine; XA: xanthine. (**c**) Shown are total glutathione (GSH + 2GSSG) level, GSH/GSSG ratio, lactate/pyruvate ratio, G6P/R5P ratio, glutamine/2-OG ratio, total Glu-related amino acids, malate/aspartic acid ratio, and total oxaloacetate-related amino acid level comparing non-treated (*blue*) and IS-treated (*red*) C2C12 cell extracts. Data are mean ± SD (*n* = 3), *p < 0.05, **p < 0.01, ***p < 0.0001, difference with control by Welch *t*-test. Vertical axis shows absolute value (pmol/10^6^ cells).

We investigated the effects of IS on intracellular metabolic alterations in skeletal muscle using C2C12 myotubes. Metabolic alterations in C2C12 myotubes treated with or without IS were analyzed using targeted metabolomics with capillary electrophoresis–mass spectrometry. Figure 3a shows the heat map of 116 targeted metabolites (Supplemental Table 2) in C2C12 myotube cells. We found that exposure to IS significantly increased cellular metabolites of the glycolysis pathway and antioxidative response-related pathways, such as the pentose phosphate pathway (PPP), and glutathione metabolism. Exposure to IS significantly decreased cellular metabolites of energy generation-related pathways, such as the tricarboxylic acid (TCA) cycle, and glutamine and glutamate.

To investigate alterations in individual metabolites with exposure to IS, we analyzed the amount of each metabolite in C2C12 myotubes (Fig. 3b). Consistent with heat map analysis, exposure to IS for 24 h significantly upregulated intermediate metabolites in the glycolysis pathway (3-phosphoglyceric acid, 2-phosphoglyceric acid), the PPP (6-phosphogluconic acid, ribulose 5-phosphate, ribose 5-phosphate (R5P), xylulose 5-phosphate, sedoheptulose 7-phosphate), glutathione metabolism (reduced glutathione (GSH), oxidized glutathione (GSSG)), and anaerobic metabolism (lactate and phosphoenolpyruvate). In contrast, exposure to IS significantly decreased intermediate metabolites in the TCA cycle (2-oxoglutarate, fumarate, and malate) and amino acids such as glutamine and glutamate.

We performed parametric analysis to confirm the metabolic alterations induced by IS exposure (Fig. 3c). In IS-treated cells, an increased total glutathione level (GSH + 2GSSG) (p < 0.01) and decreased GSH/GSSG ratio (p < 0.01) indicated intracellular oxidative stress, an increased lactate/pyruvate ratio (p < 0.05) indicated accelerated anaerobic metabolism, an increased tendency of glutamine/2-oxoglutarate ratio (p = 0.127) suggested accelerated amino acid degradation, a decreased G6P/R5P ratio (p < 0.05) indicated accelerated PPP pathway, and decreased total glutamine-related amino acids (Arg + Gln + Glu + His) (p < 0.05), oxaloacetate-related amino acids (Asn + Asp) (p < 0.05), and malate/Asp ratio (p < 0.05) indicated suppressed catabolism of amino acid to TCA-related metabolites.

Collectively, from metabolomic analyzes, we determined that IS upregulated glycolysis, PPP and glutathione metabolism and, conversely, downregulated the TCA cycle and glutamate anabolism in C2C12 myotubes.

### Alterations in glycolytic and oxidative phosphorylation activity by IS in myotubes

To evaluate the effects of IS on metabolic functions in skeletal muscle, we performed real-time monitoring of mitochondrial function and glycolytic metabolism using a flux analyzer.

**Figure 4:**
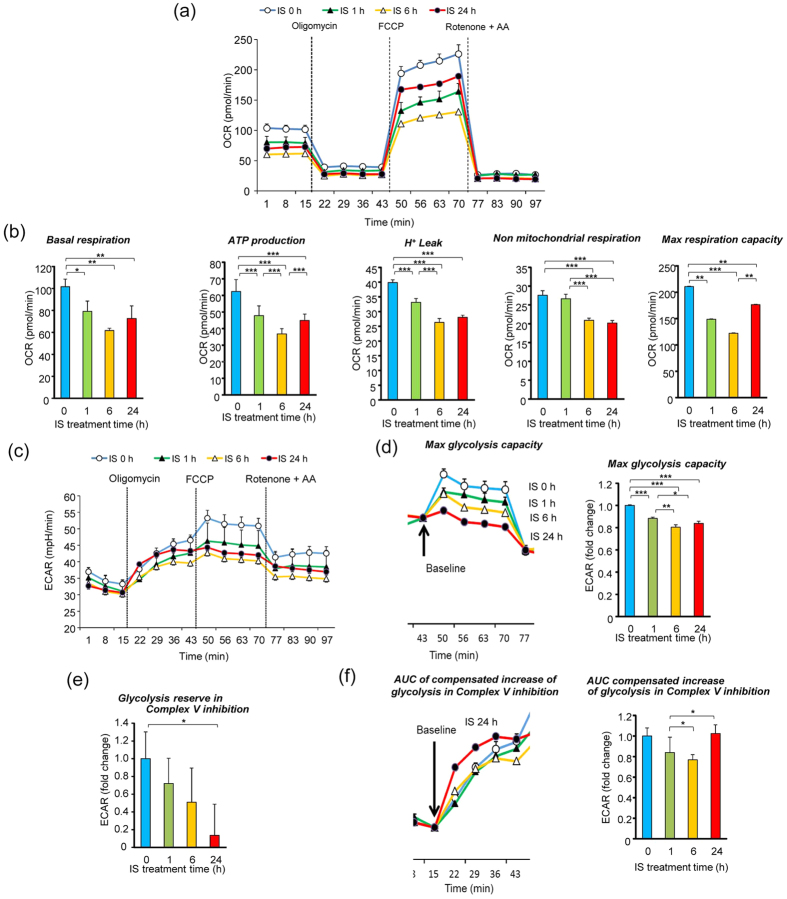
Real-time analysis of bioenergetics pathway in C2C12 myotube cells by perturbing them with small molecule metabolic modulators. Oligomycine (1 μM), carbonyl cyanide-4-(trifluoromethoxy)phenylhydrazone (FCCP) (2 μM), rotenone and antimycin A (AA) (1 μM) were injected sequentially at indicated time points into each well containing C2C12 myotube cells after baseline rate measurements. Mitochondrial function and glycolysis in C2C12 myotube cells treated with and without 1 mM IS for 24 h, as assessed by cellular oxygen consumption rate (OCR) and extracellular acidification ratio (ECAR). OCR and ECAR measurements are defined in Supplementary Fig. 2c. Shown are representative time course data for OCR **(a)** and aggregate data **(b)**. **(c)** Shown are representative time course data for ECAR. Data are mean ± SD (*n* = 3). **(d)** Shown are representative time course data (43–77 min) fit at baseline at 43 min and aggregated data. The area means max glycolysis capacity in C2C12 cells treated with IS for different times. **(e)** Aggregated data of glycolytic reserve in Complex V inhibition. **(f)** Shown are representative time course data (14–43 min) fit at baseline at 15 min and aggregated data. The area means AUC of compensated increase of glycolysis in Complex V inhibition in C2C12 cells treated with IS for different times. *p < 0.05, **p < 0.01, ***p < 0.0001, difference with control (IS: 0 h), Tukey–Kramer test. Data are mean ± SD.

A schematic illustration of the biochemical pathway and mitochondrial electron transport chain are shown in Supplemental Figs 1 and 2. Cellular oxygen consumption rate (OCR) was measured to assess electron transport chain activity and the rate of ATP production in mitochondria. To assess the specific contributions of ATP synthesis and electron transport chain activity to altered OCR, we monitored real-time OCR following sequential addition of oligomycin, carbonyl cyanide-4-(trifluoromethoxy)phenylhydrazone (FCCP), rotenone, and antimycin A. Figure 4a shows the time course for cellular OCR in C2C12 myotubes after addition of IS for 0, 1, 6, and 24 h. Exposure to IS significantly decreased basal respiration(p < 0.01), ATP production (p < 0.001), H^+^ leaking (p < 0.001), non-mitochondrial respiration (p < 0.001), and max respiration capacity (p < 0.001) time-dependently over 6 h (Fig. 4b). Interestingly, the decrease in ATP production and max respiration capacity in C2C12 myotubes with IS exposure recovered at 24-h IS exposure compared with 6-h IS exposure. Figure 4c shows the time course for extracellular acidification rate (ECAR) in C2C12 myotubes after addition of IS. Max glycolysis capacity significantly decreased with IS exposure in a time-dependent manner (p < 0.001) (Fig. 4d). Glycolytic reserve in Complex V inhibition significantly decreased in IS-treated cells (p < 0.05) (Fig. 4e). Area under the curve (AUC) of compensated increase of glycolysis in Complex V inhibition (addition of oligomycin) decreased with IS exposure time-dependently over 6 h. Similar to the time course for cellular OCR, the decreased AUC for compensated increase of glycolysis in Complex V inhibition recovered up to control levels at 24-h IS exposure (Fig. 4f). These results indicated that IS impaired mitochondrial function and glycolysis time-dependently over 6 h, following which, glycolysis was upregulated to compensate for impaired mitochondrial ATP production at 24 h.

### The PPP was upregulated via the Nrf2 pathway in muscle cells

We examined what component was responsible for upregulating glycolysis in response to 24-h IS exposure. In metabolomics analysis, PPP-related metabolites significantly increased in IS-treated C2C12 myotubes (Fig. 3b). In the PPP, NADPH production is upregulated under oxidative conditions. Generation of a reducing cofactor in the cytosol such as NADPH contributes to the elevation of ECAR, which shows upregulation of glycolysis. We therefore thought that upregulation of the PPP caused elevation of ECAR in response to IS.

**Figure 5:**
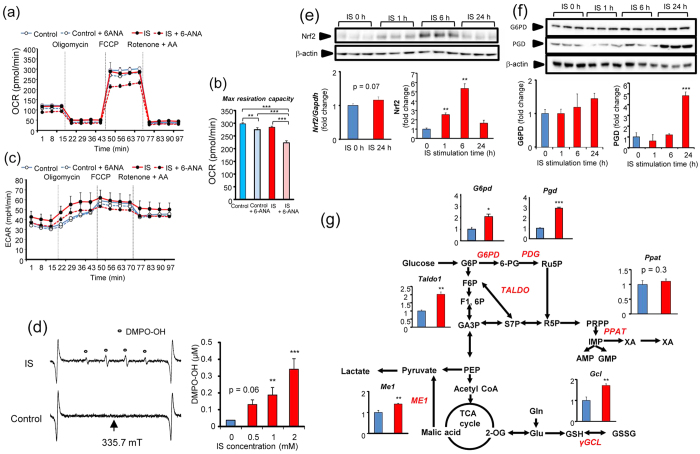
Muscle cells represents antioxidative response induced by IS through Nrf2 activation. **(a,c)** are representative time course data for ORC and ECAR in C2C12 cells treated with and without 1 mM IS for 24 h in the presence or absence of 6-aminonicotinamide (6-ANA), which is an inhibitor of the PPP, respectively. **(b)** Aggregated data of max respiration capacity. **(d)** Representative ESR spectrum of IS-treated C2C12 cultured medium. ESR spectrum shows typical hydroxyl radical trapped by DMPO and quantitative data (mean ± SD; *n* = 3) of hydroxyl radicals trapped by DMPO. Nrf2 expression **(e)** and PDG and G6PD expression **(f)** in C2C12 myotube cells. Full-length gels were shown in Supplemental Fig. 6. (**g**) The Nrf2 target gene expression of *Pgd*, *G6pd*, Taldo, *Ppat*, *Me1*, and *γGcl* in non-treated (*blue*) and IS-treated (*red*) C2C12 myotube cells (n = 3), *p < 0.05, **p < 0.01, ***p < 0.0001, Wilcoxon test. Data are mean ± SD.

To examine the contribution of the PPP in upregulated glycolysis in response to IS, we used the flux analyzer system with an inhibitor of the PPP, 6-aminonicotinamide (6-ANA). Figure 5a shows data for OCR with and without IS in the presence or absence of 6-ANA. Although 6-ANA significantly decreased the max respiration capacity in the absence of IS, 6-ANA considerably diminished the max respiration capacity in IS-treated cells (Fig. 5a,b). Figure 5c shows ECAR with and without IS in the presence or absence of 6-ANA. Although 6-ANA did not change ECAR in the absence of IS, treatment with 6-ANA decreased ECAR in the presence of IS. These findings indicated that upregulation of the PPP was responsible for the elevation of ECAR in response to IS.

To examine the molecular mechanisms behind the upregulated PPP in response to IS, we focused on nuclear factor (erythroid-2-related factor)-2 (Nrf2) and its direct targets since Nrf2 is a main transcriptional activator in response to oxidative stress^25^, and additionally regulates expression of genes in the PPP^26^. We first examined whether IS induces oxidative stress in skeletal muscle using C2C12 myotubes and electron spin resonance (ESR) analysis, which directly detects the generation of reactive oxygen species (ROS). Figure 5d shows the ESR spectrum of the cultured medium of cells treated with and without IS. The ESR spectrum showed a typical spectrum of hydroxyl radicals trapped by 5,5-dimethyl-1-pyrroline-N-oxide (DMPO). The generation of hydroxyl radicals increased in a dose-dependent manner on exposure to IS. Hydroxyl radicals were produced constantly in culture medium from the early phase of 15 min to 6 h after the addition of IS (Supplemental Fig. 3).

We next examined expression of Nrf2 and the enzymes involved with NADPH production in the PPP, such as glucose-6-phosphate dehydrogenase (G6PD) and phosphogluconate dehydrogenase (PGD)^26^. Exposure to IS increased the protein level of Nrf2 in muscle cells to a maximum level 6 h later (Fig. 5e). Consistent with the time course for Nrf2 protein levels, heme oxidase-1 and NAD(P)H-quinone oxidoreductase-1, which are major antioxidant enzymes directly regulated by Nrf2, significantly increased in IS-treated muscle cells (Supplemental Fig. 4). Further, protein expression of G6PD and PGD also increased in IS-treated muscle cells (Fig. 5f). All genes involved with NADPH production except *PPAT* significantly increased in IS-treated muscle cells (Fig. 5g). These results suggest that Nrf2 increased as an antioxidative response against IS-induced oxidative stress, and Nrf2-regulated genes for the enzymes involved with NADPH production in the PPP were upregulated.

### Mitochondrial dysfunction by IS in muscle cells

**Figure 6:**
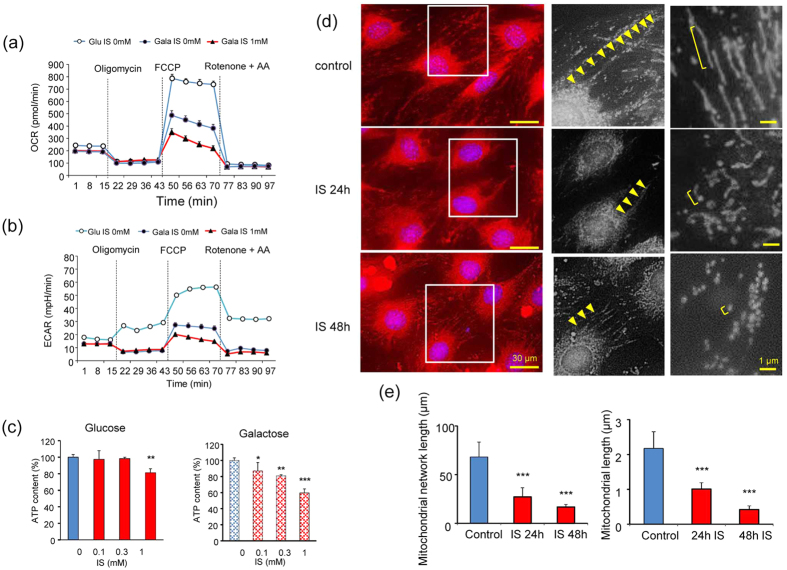
Mitochondrial alterations in C2C12 myotube cells exposure with IS. **(a,b)** are representative data for OCR and ECAR in C2C12 cells exposure with and without 1 mM IS for 24 h in glucose media or galactose media, respectively. **(c)** ATP content in C2C12 cells exposure with and without 1 mM IS for 24 h in glucose media or galactose media (n = 5). **(d)** Representative images of the mitochondrial network in C2C12 cells stained with 200 nM Mitotracker Red for 30 min and exposure with 1 mM IS for 0, 24, and 48 h. Middle columns are magnification of indicated areas in the left column. Right panels are representative magnification of mitochondria. Scale bar: 30 and 1 μm. **(e)** Quantitative analysis (mean ± SD; *n* = 10) of mitochondrial network length and mitochondrial length. *p < 0.05, **p < 0.01, ***p < 0.0001, difference with control (IS: 0 h), Tukey–Kramer’s test. Data are mean ± SD.

Exposure to IS resulted in decreased OCR, as monitoring with the flux analyzer (Fig. 4a), indicating decreased mitochondrial function in muscle cells. To assess the toxicity of IS to mitochondria in muscle cells, we cultured C2C12 cells in galactose media without glucose. Because cells cultured in galactose media are unable to perform sufficient glycolysis, they rely mostly on mitochondrial oxidative phosphorylation to generate ATP. Thus, cells cultured in galactose media are more sensitive to mitochondrial toxins than cells cultured in glucose media^27^,^28^,^29^. Muscle cells grown in galactose media showed prominently decreased max respiration capacity and glycolysis capacity compared with those grown in glucose media (Fig. 6a,b). Exposure to IS further decreased max respiration capacity and max glycolysis in muscle cells cultured in galactose compared with those cultured in glucose media. In IS-treated cells, galactose media showed an absence of the increase in the oligomycin response that was observed in glucose media (Figs 4f and 6b), indicating that the increase in the oligomycin response was derived from glycolysis, including the PPP. These results suggested that mitochondrial function was impaired with IS exposure in muscle cells, and the dysfunction further increased in the absence of glycolysis.

Additionally, we examined ATP content in both cells grown in glucose and galactose media (Fig. 6c). In the presence of IS, ATP content significantly decreased in muscle cells grown in glucose media. The decrease was prominent in muscle cells grown in galactose media. These findings suggested that mitochondrial dysfunction induced by IS leads to ATP shortage in muscle cells.

### IS causes abnormal mitochondrial morphology

Our real-time monitoring of metabolic activity indicated that IS induced mitochondrial dysfunction in muscle cells. We examined the effects of IS on the morphology of mitochondria to evaluate mitochondrial dysfunction. Although the mitochondrial network was maintained under normal culture conditions in muscle cells, it showed poor connections and increased mitochondrial fission in the presence of IS (Fig. 6d). This morphological change in mitochondria was confirmed by quantification of the network length and each mitochondrial length (Fig. 6e). These results suggested that disintegration of the mitochondrial network and increased mitochondrial fission were induced by IS in muscle cells.

### Effect of acidic conditions on metabolic alterations in muscle cells with IS exposure

**Figure 7:**
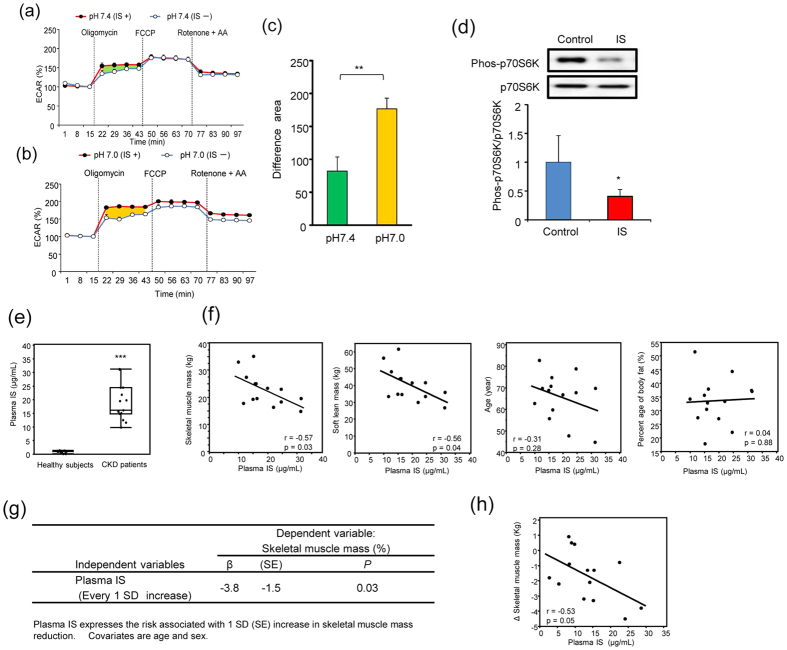
Plasma IS levels was associated with muscle mass reduction in CKD patients. **(a)** and **(b)** are representative time course data for OCR and ECAR in C2C12 cells treated with and without 1 mM IS for 24 h in pH 7.0 media or pH 7.4 media, respectively. **(c)** Difference in AUC of compensated increase of glycolysis in Complex V inhibition between pH 7.4 and pH 7.0 (n = 3), **p < 0.01, Wilcoxon test. Data are mean ± SD. **(d)** Comparison of phosphorylation levels of p70S6K between non-treated (*blue*) and IS-treated (*red*) C2C12 cells. Cells were stimulated with insulin for 30 min before harvest. Data are mean ± SD (*n* = 3), *p < 0.05, difference with control, Wilcoxon test. Full-length gel was shown in Supplementary Fig. 7. **(e)** Comparison of plasma IS level between healthy controls (n = 16) and CKD patients (n = 14). Student’s t-test. ***p < 0.001. **(f)** Single linear regression analysis between plasma IS levels and skeletal muscle mass, soft lean mass, age, and percentage of body fat. Pearson’s correlation coefficient. **(g)** Multivariable analysis between plasma IS concentration and skeletal muscle mass. **(h)** Relationship between plasma IS levels at the start of dialysis and skeletal muscle mass change over 2 years. Pearson’s correlation coefficient.

In advanced CKD, accumulation of IS causes metabolic acidosis and leads to acidification of biological fluid, processes associated with muscle wasting^14^,^15^. We performed experiments using a flux analyzer under normal conditions (pH 7.4) and pathologically acidic conditions (pH 7.0). Figure 7a,b shows representative ECAR under pH 7.4 and pH 7.0 in muscle cells treated with or without IS for 24 h. Although upregulation of glycolysis after the addition of oligomycin increased in the presence of IS under both under pH 7.4 and pH 7.0, at pH 7.0, upregulation of glycolysis after the addition of oligomycin was more severe than that at pH 7.4 (Fig. 7c) (p < 0.01). These results indicated that metabolic alterations by IS enhanced under pathologically acidic conditions.

### IS inhibits the muscle protein synthesis pathway in muscle cells

Insulin signaling is essential to the protein synthesis pathway in muscle tissue via activation of phosphatidylinositol 3-kinase/p70S6 kinase (p70S6K)^30^. To examine the effect of IS on insulin signaling in muscle cells, we evaluated phosphorylation levels of p70S6K, which is a marker for muscle protein synthesis through insulin signaling. The phosphorylation level of p70S6K reached a maximum at 30 min after insulin treatment (Supplemental Fig. 5), and significantly (p < 0.05) decreased with IS exposure to about half that of the phosphorylation level in the absence of IS (Fig. 7d). These results suggest that IS inhibits muscle protein synthesis though insulin signaling in muscle cells.

### Plasma IS levels in CKD patients are associated with skeletal muscle mass reduction

We clinically examined the association between plasma IS levels and muscle mass in CKD patients undergoing peritoneal dialysis (*n* = 14) (Supplemental Table 1). Figure 7e shows a comparison of plasma IS levels between healthy controls and CKD patients. Plasma IS levels of healthy controls and CKD patients were 0.88 ± 0.44 and 18.8 ± 6.8 μg/mL, respectively, demonstrating that plasma IS levels were significantly (p < 0.01) elevated in CKD patients. Among CKD patients, we found a significant negative relationship between plasma IS levels and skeletal muscle mass (r = −0.57, p < 0.05) and soft lean mass (r = −0.56, p < 0.05) (Fig. 7f). Age (r = −0.31, p = 0.28) and percentage body fat (r = −0.04, p = 0.88) did not show a significant correlation. Multivariable analysis suggested that plasma IS levels were independently associated with decreased skeletal muscle mass (β = −3.8, p = 0.03) (Fig. 7g). Plasma IS levels at the start of dialysis were significantly associated with the reduction in skeletal muscle over 2 years (Fig. 7h). These results indicated that accumulated IS was associated with muscle reduction in CKD patients.

## Discussion

**Figure 8:**
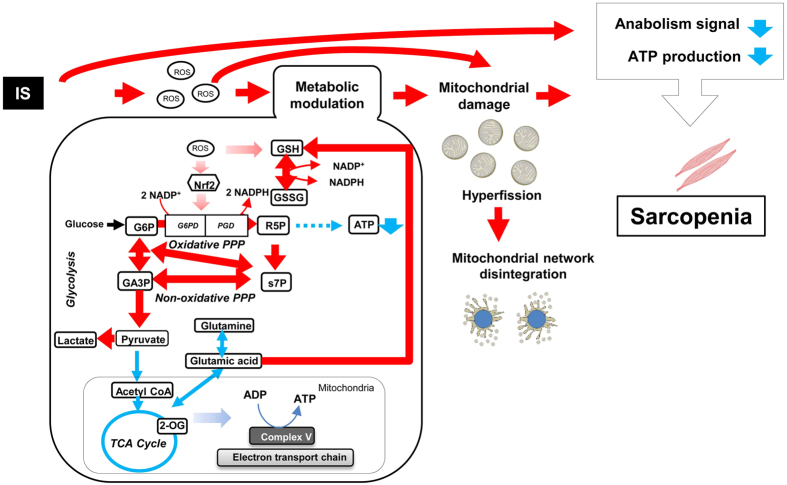
Schematic illustration of metabolic alteration induced by indoxyl sulfate in muscle. Indoxyl sulfate induces metabolic alterations such as metabolic flow changes to upregulation of antioxidative responses related Nrf2 activation, downregulation of energy-generation related pathways in muscle cells. These metabolic alterations are resulted in mitochondrial dysfunction such as mitochondrial network disintegration. Mitochondrial dysfunction caused by metabolic alteration induces suppression of anabolism signaling and ATP production, and lead to sarcopenia.

In the current study, we found that IS induced metabolic alterations in muscle cells as an antioxidative response, and these altered metabolic flow to excess antioxidative response contribute to uremic sarcopenia in CKD. A novel finding of the study is that IS induced mitochondrial network disintegration thorough metabolic alterations, such as metabolic flow changes to upregulation of antioxidative responses (PPP and glutathione metabolism), related to Nrf2 activation, and downregulation of energy-generation related pathways (TCA cycle, glutamine metabolism, and mitochondrial oxidative phosphorylation) in muscle cells, which resulted in reduced ATP production (Fig. 8).

Although inflammation has been reported as an important factor in muscle wasting in CKD^31^,^32^,^33^, inflammatory cytokines such as *IL-6* expression did not increase with IS exposure in our *in vitro* study (data not shown). Therefore, we evaluated pathogenic mechanisms of uremic sarcopenia other than inflammatory pathways.

IS accumulation in muscle cells induces oxidative stress and metabolic alterations and can result in a loss of energy production and mitochondrial network disintegration. We found that ROS production increased in IS-treated muscle cells at a similar time course to alterations in glutathione metabolism, a major scavenger of oxidative stress.

In glutathione metabolism, GSSG is reduced to GSH by GSSG reductase at the expense of NADPH. In the current study, a substantial amount of glutamine was directed into GSH synthesis, and mRNA expression of γ*GCL*, which is a rate limiting enzyme of glutathione synthesis^34^, increased with IS exposure. Expression of the NADPH production pathway, including G6P and PGD, which are targets of Nrf2^26^, also increased with IS exposure.

Our results indicated that upregulation of PPP and decreased TCA cycle are the main causes of ATP shortage in muscle cells with IS exposure. The PPP is a glucose oxidation pathway without oxygen consumption and ATP generation. Its major functions are the production of NADPH for protection against oxidative damage, and synthesis of R5P for nucleotide and nucleic acid synthesis^35^. The flow of G6P through the PPP or glycolysis is dependent on cellular requirements for NADPH, R5P, and ATP^35^. In the current study, oxidative and non-oxidative PPP increased in IS-treated muscle cells. The oxidative PPP generates NADPH and the non-oxidative PPP forms a reversible link between the PPP and glycolysis. Our results indicated that cellular requirements for NADPH increased with IS-induced oxidative damage in muscle cells, and the cells altered the metabolic flow to adjust the cellular requirements through PPP activation.

Skeletal muscle is highly metabolic and requires vast quantities of mitochondria for ATP production, as seen in the heart, liver, and kidney, making it particularly prone to xenobiotic-induced mitochondria toxicity^36^,^37^,^38^. Our data showed downregulation of the TCA cycle, reduction of ATP production and mitochondrial function, and induction of mitochondrial network disintegration in IS-treated muscle cells. Decreased ATP content in muscle cells grown in galactose media was observed, and cells cultured in galactose media were unable to upregulate glycolysis (ECAR) and maintain sufficient ATP levels following inhibition of OCR by IS-induced mitochondrial toxicity in this study. These results suggest that IS induces mitotoxicity and leads to ATP shortage in muscle cells.

Although sarcopenia is present in a large proportion of CKD patients and is a risk factor for mortality in patients undergoing dialysis treatment^3^,^4^, the role of IS in skeletal muscle and mitochondrial dynamics in the progression of sarcopenia is not yet fully understood. Our results suggest the possibility that IS contributes to muscle mass without altering fat mass and progresses sarcopenia through mitochondrial network disintegration.

The association between plasma IS and skeletal muscle mass has never been examined, although plasma uremic toxins accumulate in CKD patients. We found that plasma IS levels are associated with muscle mass reduction, but not age and percentage of fat volume. Especially, the association between plasma IS levels at the start of dialysis and muscle mass change over 2 years is new perception. These results suggest that IS is a factor inducing sarcopenia in CKD patients.

Loss of muscle mass and cachexia have been observed in a number of disease states, such as cancer^39^, AIDS^39^,^40^, and diabetes mellitus^40^,^41^. Cachexia is a wasting syndrome associated with elevated basal energy expenditure and loss of adipose and muscle tissue, like sarcopenia. Cancer cells consume large quantities of nutrients, such as glucose and glutamine, and shunt the metabolites into the anabolic pathway, and their proliferation is accelerated via PPP activation induced by Nrf2^26^. We also found that IS exposure resulted in decrease of glutamine and upregulation of PPP through Nrf2 activation. Therefore, it is possible that metabolic alterations, such as excess upregulation of PPP, is a cause of increased basal energy expenditure leading to muscle mass loss in various diseases.

Klotho is an anti-aging gene, and secreted Klotho protein protects cells and tissues from oxidative stress^42^. Recently some studies have shown that IS reduces Klotho expression by activating nuclear factor-kB through production of ROS in the kidney^43^,^44^. In addition, some studies have shown the clinical relationship between Klotho levels and physiology of skeletal muscle^43^,^45^,^46^. Taken together, it is possible that downregulation of Klotho by IS in muscle tissue is also a mechanism of muscle mass loss in CKD.

Our work suggests that metabolic alterations and mitochondrial disintegration induced by the uremic toxin IS are critical causes of uremic sarcopenia, and targeting these alterations may be useful for the prevention or treatment of uremic sarcopenia. To validate the findings obtained in the cell line in the present study, further studies would be required to examine whether the similar metabolic changes are observed in muscle tissues of CKD model animals and of CKD patients, if possible.

## Methods

### Animal studies

All animal experiments were approved by the Animal Committee of Tohoku University School of Medicine. Experimental protocols and animal care were performed according to the guidelines for the care and use of animals established by Tohoku University. Male C57BL/6 mice were fed a normal diet. At 7 weeks old, mice were randomly divided into control and adenine groups. The control group was continued on the normal diet and the adenine group was fed a diet containing 0.2% adenine (Wako) for 6 weeks. After 6 weeks, mice were killed and tissues obtained.

### Study population

Fourteen peritoneal dialysis patients were recruited from Tohoku University Hospital. Patients were subjected to peritoneal equilibration tests (PET) more than three times after peritoneal dialysis treatment started. There were eight men and six women with a mean age of 65.0 years (range: 44–83 years). Primary diseases were diabetic nephropathy, hypertension, chronic glomerulonephritis, or IgA nephropathy. At each PET, plasma and peritoneal effluent were collected. All samples were stored at −80 °C until analysis. Sixteen healthy participants (eight men and eight women, aged 32.9 ± 7.0 years) were enrolled as the control group. The study protocol was approved by the Institutional Ethical Review Board of Tohoku University School of Medicine, and was conducted in accordance with the principles contained in the Declaration of Helsinki. Informed consent was obtained from all patients. Participant information including levels of IS in plasma and peritoneal dialysate concentration are presented in Supplemental Table 1.

### Cell cultures

Mouse C2C12 myoblast cell line was obtained from the American Type Culture Collection (Manassas, VA, USA) and grown in DMEM (Gibco), containing 10% fetal bovine serum, 100 IU/mL penicillin and 100 IU/mL streptomycin in a humidified incubator at 37 °C with 5% CO_2_ in air. Confluent C2C12 myoblasts were differentiated into myotubes by incubation with DMEM containing 2% horse serum for 4–5 days. After differentiation, C2C12 myotubes were incubated with media containing 4% bovine serum albumin and different concentrations of IS for 24 h. After 24-h incubation, C2C12 myotubes were stimulated with 100 nM insulin for 60 min in the presence or absence of IS. C2C12 myotubes were then treated for each analysis.

### Imaging mass spectrometry of muscle tissue sections

Muscle tissues were sectioned at 10-μm thickness with a cryostat and thaw-mounted onto ITO-coated glass slides. 9-AA (600 mg) was deposited on slides at 0.5-μm thickness in an iMLayer (Shimadzu, Kyoto, Japan), and recrystallization was carried out by the methods described previously with slight modification^47^. In this study, Matrix Assisted Laser Desorption/Ionization Time-of-Flight Mass Spectrometer (MALDI-TOFMS, AXIMA^®^ Confidence, Shimadzu) equipped with a 337 nm N_2_ laser was used for mass spectrometry analysis. Mass spectra were acquired with the laser frequency in the negative and scanning mass range from *m/z* 50 to *m/z* 1,000 at a high-resolution mode. Laser power, detection voltage, and accumulated number of MALDI-TOFMS were 115, 3.0 kV, and 1/pixel, respectively. The spatial interval of data points was 50 μm, giving 14641 profiles in total for the section. Data collected through the microscopic system were digitally processed using Imaging MS Solution analysis software (Shimadzu). The signal intensity of each imaging data in the figure is represented as the normalized intensity. Metabolites were identified with the MS/MS spectrum from results of IS chemical standard analysis using MALDI-QIT-TOFMS (AXIMA^®^ QIT, Shimadzu).

### Sample preparation for LC-MS/MS measurement

One hundred and fifty μL of 0.1% formate methanol was added to 50 μL of sample (plasma, PD fluid, cell culture medium, or cell lysate), and vortexed for 1 s. After vortexing, samples were sonicated for 5 min, and centrifuged at 16 400 × *g* for 20 min at 4 °C. The supernatant was filtered through membranes (pore size: 0.22 μm; Merck Millipore, Billerica, MA, USA) and analyzed by LC-MS/MS.

### Analytical measurements

Quantitative analysis of IS using LC-MS/MS was performed using a Prominence LC system (Shimadzu) coupled to a TSQ Quantum-Ultra (Thermo Fisher Scientific, San Jose, CA, USA) and operated in the negative mode. Each sample (4 μL) was injected onto a 100 × 2.0 mm SeQuant ZIC-HILIC (Merck Schuchardt) with a flow rate of 0.4 mL/min. For gradient elution, mobile phase A was H_2_O/acetonitrile/HCOOH = 98/2/0.1 and mobile phase B was H_2_O/acetonitrile/HCOOH = 2/98/0.1. Linear and stepwise gradients were programmed as follows: 0–1 min: 10% solvent B; 1.1–2 min: 10–55% solvent B; 2.1–4 min: 55–90% solvent B; 4.1–6 min: 90–100% solvent B; 6.1–11 min: 100% solvent B; 11.1–15 min: 10% solvent B. Bilirubin and biliverdin-d_4_ were detected in selected reaction monitoring mode by monitoring the transitions of *m/z* 212 to 80 and 216 to 80, respectively. Spray voltage was 3 500 V, vaporizer temperature was 275 °C, and ion transfer tube temperature was 350 °C. To validate each assay, the followings were performed. Intra and inter assay coefficients of precision were 4.03% and 0.049% at 0.2 μg/mL, respectively, and intra and inter assay coefficients of accuracy were 8.62% and 3.1% at 0.2 μg/mL, respectively. Calibration curve was prepared in every batch, and the correlation coefficient was over 0.999.

### Cell viability

To assess the effects of IS on skeletal muscle cell growth and viability, 3-(4, 5-dimethylthiazol-2-yl)-2,5-diphenyltetrazolium bromide (MTT) assay (10009365; Cayman Chemical Co., Ann Arbor, MI, USA) was performed according to the manufacturer’s protocol. Cells seeded in 96-well plates were treated with different concentrations of IS for 24 and 48 h.

### Seahorse XF24 extracellular flux measurements

Mitochondrial respiration was assessed using a Seahorse XF24 Extracellular Flux Analyzer (Seahorse Bioscience, North Billerica, MA, USA). C2C12 myoblast cells were seeded on Seahorse XF-24 plates at a density of 2.0 × 10^3^ cells/well. C2C12 myoblast cells were differentiated to myotubes as described above. Cells were treated with 1 mM IS or 50 mM Tris-HCl for 24 h. One day prior to the experiment, sensor cartridges were hydrated with XF calibrate solution (pH 7.4) and incubated at 37 °C in a non-CO_2_ incubator for 24 h. Before assessment, each well was washed with DMEM solution supplemented with 5.6 mM glucose, 1 mM sodium pyruvate, 32 mM NaCl, 2 mM GlutaMax (pH 7.4) and incubated at 37 °C for 30 min. Baseline measurements of OCR and ECAR were taken before sequential injection of the following inhibitors: 1 μM oligomycin, which is an ATP synthase inhibitor; 2 μM FCCP, which is a mitochondrial respiration uncoupler; and 1 μM antimycin A and rotenone, which are mitochondrial electron transport blockers. Oligomycin was applied first to estimate the proportion of basal OCR coupled to ATP synthesis. After oligomycin application, FCCP was used to further determine maximal glycolysis pathway capacity.

### ATP assay

Cellular ATP production was assessed using a Luminometric ATP assay kit (Toyo B-Net, Tokyo, Japan). Briefly, cells were treated with IS for 24 h in glucose or galactose media before 100 μL/well of ATP reaction mixture was added to the sample and mixed gently. The plate was incubated at 23 °C for 10 min in the measurement machine. Luminescence intensity was detected at 23 °C.

### Mitochondrial dynamics analysis

Cells were incubated for 30 min with 200 nM Mitotracker Red CMXRos (Invitrogen). An all-in-one fluorescence microscope (BZ-X710; Keyence, Osaka, Japan) was used to obtain images.

### Statistical analyses

Values are reported as mean ± standard deviation (SD) unless otherwise indicated. Data were analyzed using JMP software version 11 (SAS Institute Inc., Cary, NC, USA). Student *t*-test, Welch *t*-tests, Wilcoxon test, Dunnett’s tests, and Tukey–Kramer’s tests were used for two variables comparisons and multivariate comparisons, as appropriate.

## Additional Information

**How to cite this article**: Sato, E. *et al*. Metabolic alterations by indoxyl sulfate in skeletal muscle induce uremic sarcopenia in chronic kidney disease. *Sci. Rep*. **6**, 36618; doi: 10.1038/srep36618 (2016).

**Publisher’s note:** Springer Nature remains neutral with regard to jurisdictional claims in published maps and institutional affiliations.

## Supplementary Material

Supplementary Information
