# Supplementary material for: Metabolic alterations by indoxyl sulfate in skeletal muscle induce uremic sarcopenia in chronic kidney disease
Source: Sci Rep. 2016 Nov 10;6:36618. doi: 10.1038/srep36618 (PMC5103201; doi:10.1038/srep36618)
Supplement: Supplementary Information [file srep36618-s1.docx]

**Supplementary information**

**Metabolic alterations by indoxyl sulfate in skeletal muscle induce uremic sarcopenia in chronic kidney disease**

Emiko Sato^1,2^, Takefumi Mori^1,3^, Eikan Mishima^1^, Arisa Suzuki^1^, Sanae Sugawara^1^, Naho Kurasawa^1^, Daisuke Saigusa^4^, Daisuke Miura^5^, Tomomi Morikawa-Ichinose^5^, Ritsumi Saito^4^, Ikuko Oba-Yabana^1^, Yuji Oe^1^, Kiyomi Kisu^1^, Eri Naganuma^1^, Kenji Koizumi^1^, Takayuki Mokudai^6^, Yoshimi Niwano^6^, Tai Kudo^7^, Chitose Suzuki^1^, Nobuyuki Takahashi^1,2^, Hiroshi Sato^1,2^, Takaaki Abe^1^, Toshimitsu Niwa^8^, Sadayoshi Ito^1^

^1^ Division of Nephrology, Endocrinology and Vascular Medicine, Tohoku University Graduate School of Medicine, Sendai 980-8574, Japan

^2^ Division of Clinical Pharmacology and Therapeutics, Tohoku University Graduate School of Pharmaceutical Sciences, Sendai 980-8578, Japan

^3^ Division of Integrative Renal Replacement Therapy, Tohoku University Graduate School of Medicine, Sendai 980-8574, Japan

^4^ Department of Integrative Genomics, Tohoku Medical Megabank Organization, Tohoku University, Sendai 980-8573, Japan

^5^ Innovation Center for Medical Redox Navigation, Kyushu University, Fukuoka 812-8582, Japan

^6^ Laboratory for Redox Regulation, Tohoku University Graduate School of Dentistry, Sendai 980-8575, Japan

^7^ Primetech Co. Ltd., Tokyo 112-0002, Japan

^8^ Faculty of Health and Nutrition, Shubun University, Ichinomiya 491-0938, Japan

**Supplementary methods**

**Materials**

IS and Dulbecco’s Modified Eagle’s Medium (DMEM) were purchased from Sigma Aldrich (St. Louis, MO, USA), C2C12 was purchased from American Type Culture Collection (Manassas, VA, USA), 3-indoxyl sulfate-d4 was purchased from Toronto Research Chemicals (Toronto, Canada), 9-aminoacridine (9-AA) was purchased from Merck Schuchardt (Hohenbrunn, Germany).

**Quantitative capillary electrophoresis–mass spectrometry metabolite profiling**

Capillary electrophoresis–mass spectrometry-based targeted quantitative analysis was performed at Human Metabolome Technologies, Inc. (Yamagata, Japan). A total 116 metabolites involved in glycolysis, PPP, TCA cycle, urea cycle, and polyamine, creatinine, purine, glutathione, nicotinamide, choline, and amino acid metabolism were analyzed (Supplemental Table 2). To measure the levels of metabolites in the C2C12 myotube cells, an aliquot (675 μL) of methanol containing internal standards was added to 1 × 10^6^ cells to extract cellular metabolites.Resultant cellular extract was centrifuged at 2,300 ×g for 5 min at 4ºC. Subsequently, 175 μL of upper aqueous layer was centrifugally filtered through a Millipore 5-kDa cutoff filter at 9,100 ×g for 120 min at 4ºC to remove proteins. The filtrate was used for analysis. The measurement conditions were identical to those reported previously^1-3^. Cationic compounds were measured in the positive mode of CE-TOFMS (Agilent CE-TOFMS system Machine No.3, Fused silica capillary, i.d. 50 μm × 80 cm), and anionic compounds were measured in the positive and negative mode of CE-MS/MS (Agilent CE system and Agilent 6400 TripleQuad LC/MS Machine No. QqQ1, Fused silica capillary, i.d. 50 μM × 80 cm). Peaks detected by CE-TOFMS and CE-MS/MS were extracted using automatic integration software (MasterHands, Keio University, Tsuruoka, Japan) and Mass Hunter Quantitative Analysis B.06.00 (Agilent Technologies, SantaClara, CA, USA) in order to obtain peak information including m/z, migration time (MT), and peak area. The peaks were annotated with putative metabolites from the HMT metabolite database based on their MTs and m/z values determined by CE and TOFMS, respectively. The tolerance range for the peak was configured at ±0.5 min for MT and ±10 ppm for m/z. Concentrations of metabolites were calculated by compensating the peak area of each metabolite with the area of the internal standard.

**PCR analysis**

Total RNA was extracted using an RNeasy Mini kit (Qiagen, Hilden, Germany) according to the recommended protocol. Extracted RNA was reverse transcribed to cDNA using SuperScript III First-standard Synthesis SuperMix (Invitrogen, Carlsbad, CA, USA) according to the recommended protocol. PCR was performed in a total volume of 25 µL containing aliquots of cDNA, 10 µM of each primer, and SYBER Premix Taq II (Takara, Kusatsu, Japan). After heating at 94°C for 2 min, denaturation, annealing, and elongation were carried out at 95°C for 30 s, 95°C for 5 s, and 60°C for 20 s, respectively. Reactions were repeated for 39 cycles. Expression of glyceraldehyde-3-phosphate dehydrogenase (*GAPDH*) mRNA was used as an internal control. Each gene of interest (*NFE2L2, G6PD2, PGD, ME1, NQO1, TALDO1, HMOX1, GCLC,* and *PPAT*) were obtained from Takara and information is shown in Supplemental Table 2.

**Western blotting**

Protein was extracted using 1 × RIPA buffer (Cell Signaling Technology, Danvers, MA, USA) containing a protease inhibitor (Roche Diagnostics K.K, Tokyo, Japan), phosphatase inhibitor cocktail (Sigma Aldrich), and 1 mM PMSF (Thermo Scientific, Waltham, MA, USA). With Nrf2 protein extraction, 10 μM MG132 (Sigma Aldrich) was additionally added to the above extraction buffer. Protein concentration was determined using a Quick Start protein assay (Bio-Rad Laboratories, Hercules, CA, USA), and 25 μg of protein was used in each SDS-PAGE run. 7.5% Mini-Pro TEAN Precast Gel (Bio-Rad) was used for each analysis. Protein extracts were transferred to a PVDF membrane. After blocking for 1 h, the membrane was incubated with primary antibodies (anti-NRF2, 1:200, #14596, Cell Signaling; anti-p70S6 Kinase, 1:1000, #9202, Cell Signaling; anti-Phospho p70S6 Kinase, 1:1000, #9205, Cell Signaling; anti-PGD, 1:1000, ab96225, Abcam; anti-G6PD, 1:1000, #12263, Cell Signaling) overnight at 4ºC. After washing, the membrane was incubated with secondary antibodies (anti-rabbit IgG, 1:5000, sc-2004, Santa Cruz Biotechnology, Dallas, TX, USA; anti-mouse IgG, 1:5000, sc-2005, Santa Cruz; anti-rat IgG, sc-2032, 1:5000, Santa Cruz) for 1 h at room temperature. Expression of b-actin (1:5000, sc-47778, Santa Cruz) was used as an internal control.

**ROS detection by electron spin resonance**

Free radical species produced by C2C12 were measured by ESR analysis as described previously^4^. C2C12 myoblast cells (5 × 10^5^ cells/well) were seeded and differentiated into myotubes. C2C12 myotubes were washed with Dulbecco’s phosphate-buffered saline and incubated in the same solution containing 5 mM D-glucose for 30 min at room temperature. Cells were incubated in 600 μL of Dulbecco’s phosphate-buffered saline containing 5 mM D-glucose and various concentrations of IS at 37°C for 1 h. Following incubation, 30 μL of DMPO at 2.97 M was added and incubated for another 10 min. The medium was then measured using an ESR spectrometer (JES-FA-100; Jeol, Tokyo, Japan). ESR settings were microwave frequency: 9.4 GHz; microwave power: 8.0 mW; time constant: 0.03 s; sweep time: 120 s, center field: 337.1 mT; scan range: ± 5 mT; modulation frequency: 100 kHz; field modulation width: 0.1 mT; amplitude: × 800.

**Body composition measurements**

Body composition was assessed using multi-frequency bioelectrical impedance with eight tactile electrodes (InBody 720; Biospace, Tokyo, Japan). With this method, body weight, body mass index, skeletal muscle mass, and percent body fat are automatically and simultaneously measured.

**Supplemental Reference:**

1. Soga, T.*, et al.* Simultaneous determination of anionic intermediates for Bacillus subtilis metabolic pathways by capillary electrophoresis electrospray ionization mass spectrometry. *Anal Chem* **74**, 2233-2239 (2002).

2. Soga, T. & Heiger, D.N. Amino acid analysis by capillary electrophoresis electrospray ionization mass spectrometry. *Anal Chem* **72**, 1236-1241 (2000).

3. Soga, T.*, et al.* Quantitative metabolome analysis using capillary electrophoresis mass spectrometry. *J Proteome Res* **2**, 488-494 (2003).

4. Motojima, M., Hosokawa, A., Yamato, H., Muraki, T. & Yoshioka, T. Uremic toxins of organic anions up-regulate PAI-1 expression by induction of NF-kappaB and free radical in proximal tubular cells. *Kidney Int* **63**, 1671-1680 (2003).
